# Production of Flavonoids in Callus Cultures of *Sophora flavescens* Aiton

**DOI:** 10.3390/plants9060688

**Published:** 2020-05-28

**Authors:** Ji-Sun Park, Zuh-Kyung Seong, Mi-Sun Kim, Jang-Ho Ha, Ki-Beom Moon, Hyo-Jun Lee, Hyeong-Kyu Lee, Jae-Heung Jeon, Sang Un Park, Hyun-Soon Kim

**Affiliations:** 1 Plant Systems Engineering Research Center, KRIBB, 125 Gwahak-ro, Yuseong-gu, Daejeon 34141, Korea; pjs12315@kribb.re.kr (J.-S.P.); liebevoll77@kribb.re.kr (M.-S.K.); hjh0398@kribb.re.kr (J.-H.H.); irony83@kribb.re.kr (K.-B.M.); hyojunlee@kribb.re.kr (H.-J.L.); jeonjh@kribb.re.kr (J.-H.J.); 2 Department of Crop Science, Chungnam National University, 99 Daehak-ro, Daejeon 34143, Korea; 3 Natural Medicine Research Center, KRIBB, 30 Yeongudanji-ro, Ochang-eup, Cheongju-si, Chungbuk 28116, Korea; sls0486@kribb.re.kr (Z.-K.S.); hykylee@kribb.re.kr (H.-K.L.)

**Keywords:** callus induction, herbal resource, plant growth regulators, secondary metabolite, *Sophora flavescens*

## Abstract

Flavonoids, including maackiain (Maac) from *Sophora flavescens* Aiton roots, have many pharmacological properties, such as antitumor, antimicrobial, and antifungal activities. This research aimed to develop an in vitro plant and callus culture system for *S. flavescens* for the purpose of generating an alternative production system for enhancing Maac production, as Maac is usually present in very small amounts in *S. flavescens’* roots. We arranged the optimal conditions of different tissues of *S. flavescens* and supplemented the medium with various plant growth regulators (PGRs). The highest induction and proliferation rates of callus was shown in combination treatments of all concentrations of thidiazuron (TDZ) and picloram. In addition, calli induced with leaf explants cultured on 2.0 mg/L picloram and 0.5 mg/L 6-benzyladenine (BA) in Murashige and Skoog (MS) medium had the highest accumulation of the active metabolite Maac. In vitro shoots were regenerated on medium containing combinations of TDZ and α-Naphthalene acetic acid (NAA). A reliable protocol for the mass production of secondary metabolites using a callus culture of *S. flavescens* was successfully established.

## 1. Introduction

Medicinal and aromatic plants may heal and cure human diseases and have gained worldwide attention as alternative therapies due to their efficacy and safety with few associated side effects [1]. Isolated bioactive compounds such as flavonoids and phenolic and polyphenolic compounds are used directly or semi-synthetically as food additives, flavors, cosmetics, and other industrially important biochemicals [2,3]. *Sophora flavescens* Aiton, which is a diffused species from the Fabaceae family, grows widely throughout Asia. The root of *S. flavescens* has been commonly used for improvement of asthma, sores, allergies, and inflammation as well as for treating diarrhea, gastrointestinal hemorrhage, and eczema [4,5]. Flavonoids, such as prenylated or lavandulylated flavanones, and a series of lupin alkaloids from *S. flavescens’* roots have many attractive pharmacological properties, such as antitumor, antimicrobial, and antifungal activities [6,7]. Maackiain (Maac) is a flavonoid metabolite classified as a derivative of pterocarpan and is present in very small amounts in the *S. flavescens’* root.

Industries use in vitro culture systems for mass production of the bioactive compounds of medicinal plants to ensure a continuous supply in a relatively short period of time. Harvesting the roots and underground parts requires the plant to be cut down [8,9], which negatively affects rare plant species and leads to limited quantities of wild plants [10,11]. In vitro systems employ plant cell culture technology without destroying the natural habitat. Previous studies established production systems of secondary metabolites through in vitro cultures [12,13,14,15]. Callus cultures of *S. flavescens* were first established by Furuya and Ikuta [16], and the production of prenylated flavanones (sophoraflavanone G, lehmannin, and pterocarpans) and their glycosides from cell suspension cultures has been previously reported [17,18].

The balance of plant growth regulators (PGRs), especially auxin and cytokinin, has an important effect on the determination of plant regeneration through organogenesis or somatic embryogenesis from callus. In studies aimed at producing useful ingredients through callus culture, not only induction of callus but also continuous proliferation are entirely dependent on the effects of PGRs. In the latter case, a specific type and a relatively high concentration of auxin are mainly used according to the plant species. In *Nothapodytes foetida*, 16.11 μM (micromole) NAA (α-Naphthalene acetic acid) and 2.22 μM BA (6-benzyladenine) are the best PGRs’ condition for callus induction [19], otherwise, in *Allium sativum*, 4.5 μM 2,4-D and 4.43 μM BA are the best combination [20]. The effectiveness of 2,4-D, 4-amino-3,5,6-trichloropicolinic acid (picloram), benzo[b]selenienyl acetic acid (BSAA), and NAA on callus induction has been proven in many other reports [21,22,23,24]. Also, it is possible to increase the content of bioactive secondary metabolites in callus due to various culture conditions, especially the combination of types and concentrations of PGRs. In *Pyrostegia venusta*, 9.05 μM 2,4-D and 8.88 μM BAP (6-benzylamino purine) treatments resulted in obtaining calli with contents of total phenolic compounds and flavonoids [25]. *Andrographis lineata* callus culture producing Echioidinin and 7-O-Methywogonin as a source for cancer chemotherapeutic agents was established on MS (Murashige and Skoog) medium containing 1.0 mg/L IAA (Indole-3-acetic acid) [26].

This study aimed to develop an in vitro plant and callus culture system for *S. flavescens* with the objective of generating an alternative production system for isoflavonoids.

## 2. Results

### 2.1. In Vitro Germination of S. flavescens

**Figure 1:**
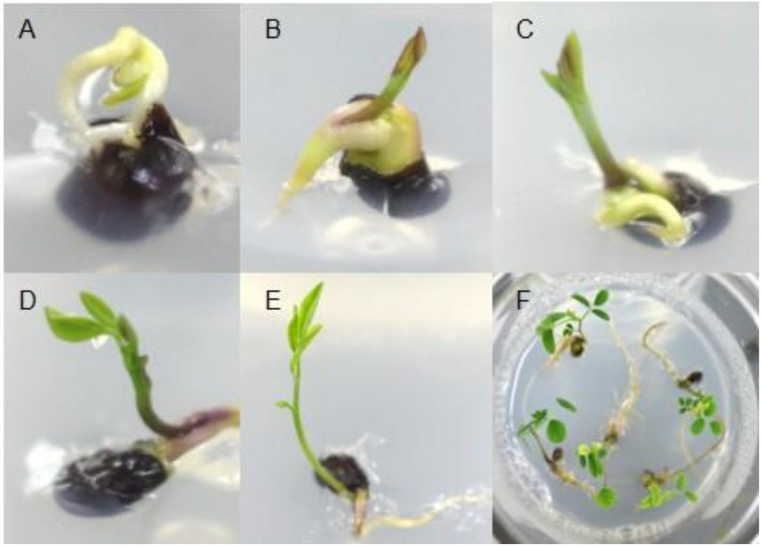
In vitro germination of *Sophora flavescens* Aiton. Three days (**A**), 5 days (**B**), 7 days (**C**), 10 days (**D**), and 14 days (**E**) after germination with root development. In vitro plant growth (**F**). Seeds were dipped in 2.0 mg/L GA_3_ (Gibberellin A_3_) for 24 h, and then the surface was sterilized. Then, they were placed on plant growth regulator-free MS (Murashige and Skoog) medium.

The first visible sign of germination appeared 3 days after sowing in the germination medium; greenish shoots and roots emerged in about 7 days and 12 days, respectively (Figure 1). One-month-old seedlings had the appearance of an intact plant with fully expanded leaves, and germination rates were about 25%. No previous studies have reported on seed germination of *S. flavescens*. In this study, we used seeds that had been stored for 2 years, which may have caused germination reduction. Norton et al. [27] assessed germination rates between 24- and 40-year-old and fresh *Sophora* seeds, and found that although *Sophora* seeds retained their viability after prolonged storage, long periods of storage dramatically decreased the germination rates compared with fresh seeds.

### 2.2. Callus Induction and Selection

A total of 100 combinations of different types and concentrations of PGRs were added to MS medium to induce calli from leaf, stem, and root segments. After 8 weeks of culture, callus initiation at the cut surfaces of explants was observed on media containing the most combinations of PGRs; the highest rate (100%) was shown for all types of explants for all concentrations of TDZ (thidiazuron) and picloram combinations (data not shown). In contrast, the PGR-free and kinetin-only cultures showed no callus formation for all types of explants. The stem and root explants were more suitable for callus induction than the leaf explants under the same conditions.

**Figure 2:**
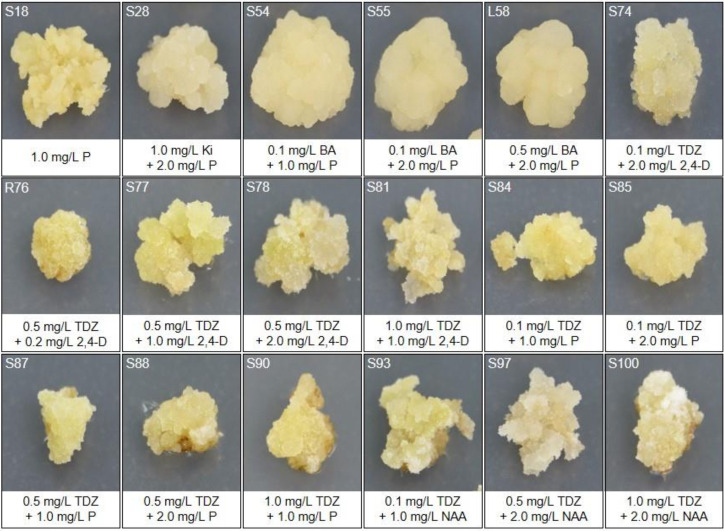
Variations in callus color and morphology under different culture conditions after 8 weeks of cultures. Conditions for each numbered callus are presented under the photographs. P, picloram; Ki, kinetin; BA, 6-benzyladenine; TDZ, thidiazuron; 2,4-D, 2,4-dichlorophenoxyacetic acid.

Induced calli were subcultured every 4 weeks in the same medium and proliferated to 5-fold each time. Representative calli derived from various PGR combinations are shown in Figure 2. It is apparent that yellow and juicy calli were formed in media combinations of BA and picloram (callus number 54, 55, and 58), and white and sticky calli were formed in TDZ and NAA combinations (callus number 93, 97, and 100). The calli induced in medium containing kinetin and picloram turned brownish after about 5–6 weeks. Otherwise, the calli induced in the medium containing BA propagated well regardless of auxin, but many calli were wet and easily aggregated. In the medium containing TDZ, the callus was transparent, yellowish, and compact.

### 2.3. Total Phenolic and Flavonoid Contents

After 6 months of subculture, we obtained one line of leaf-derived, one line of root-derived, and three lines of stem-derived calli, which were well-proliferated and had an appropriate appearance. These were used as materials for the analysis of the accumulation of secondary metabolites. The total phenolic content of the L58 callus extract, calculated from the calibration curve (R^2^ (coefficient of determination) = 0.998), was 6.00 ± 0.30 gallic acid equivalents/g, and the total flavonoid content (R^2^ = 0.999) was 5.40 ± 0.56 quercetin equivalents/g (Table 1), exhibiting a much higher phenolic and flavonoid content than other lines. In wild-type root, phenolic and flavonoid compounds were 7.82 ± 0.52 mg GAE (gallic acid equivalents)/g FW (fresh weight) and 9.75 ± 0.18 mg QE (quercetin)/g FW, respectively, accumulating higher amounts than any of the others. Phenolic compounds are one of the redox property compounds providing defense against pathogenic attack, as signaling molecules, and regulating vital biochemical processes including antioxidant activity [28]. Plant flavonoids, including flavones and flavanols, have antioxidant activity as secondary metabolites.

### 2.4. Analysis of Flavonoid Metabolites, Including Maackiain, in Calli by UHPLC

**Figure 3:**
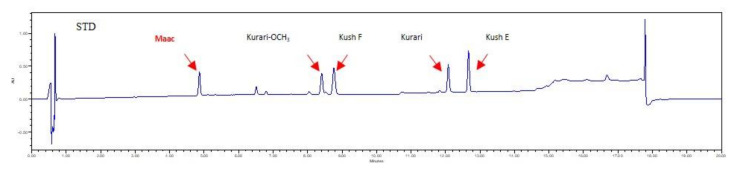
Chromatogram (total run time = 20 min) of authentic standards of Maac (4.9 min), Kurari-OCH3 (8.4 min), Kush F (8.78 min), Kurari (12.1 min), and Kush E (12.65 min). Maac, maackiain; Kurari-OCH3, methoxy kurarinone; Kush F, kushenol F; Kurari, kurarinone; Kush E, Kushenol E.

Isoflavonoid-related metabolites in the five independent calli lines were analyzed by UHPLC (Ultra-High Performance Liquid Chromatography). Five new compounds, those classified as flavanones, were characterized by comparison of their retention times with Maac (4.9 min), Kurari-OCH_3_ (8.4 min), Kush F (8.78 min), Kurari (12.1 min), and Kush E (12.65 min) (Figure 3). In accordance with the values of dry biomass, calli induced and proliferated on medium containing 2.0 mg/L picloram and 0.5 mg/L BA from leaf explants, named L58, produced the highest amount of Maac (1580.6 μg/g dry weight) (Table 2), which was 10- and 12-fold higher than that of the field-grown plant root (145.9 μg/g) and in vitro plant root (123.3 μg/g), respectively. Other root- or stem-derived calli (named as R76, S18, S55, and S88) yielded nearly similar levels of Maac between them, producing higher levels than in plant tissues (Table 2). Interestingly, field- or in vitro-grown plant root, but not calli, contained Kurari-OCH_3_ and Kurari. Kush E was not detected in any samples. These calli containing flavonoid compounds were analyzed for antioxidant activity.

### 2.5. DPPH Radical Scavenging Activity

To measure the antioxidant activity, DPPH, as a free radical, along with ascorbic acid as a control, is typically employed, followed by examination of samples for percentage of scavenging free radicals [29]. DPPH activity was detected in five different calli, WT (wild type) plant root, and in vitro plant root (Table 1). Stem calli grown on MS medium containing 1.0 mg/L picloram alone exhibited the lowest antioxidant activity (32.0%), while further increases in antioxidant activity were observed in calli grown on MS medium containing 2.0 mg/L picloram combined with cytokines, such as 0.1 mg/L BA (55.0%) or 0.5 mg/L TDZ (53.0%), respectively. The highest value was detected in the L58 calli (62.0%) grown on MS media containing 0.5 mg/L BA + 2.0 mg/L picloram. The antioxidant activity of calli was comparable to that of the roots of wild-type and in vitro-grown *S. flavescens*. A total of 84% and 70% DPPH for wild-type- and in vitro-derived roots, respectively, were higher than those of the callus cultures (Table 1).

### 2.6. POD and SOD Activities

The induction of calli is considered as a stress factor in cultivated plants. We, therefore, analyzed SOD and POD enzyme activities. Both are iso-enzymes, differentiated by various physical and chemical features and amino acid sequences, but with the same catalyst reaction. The enzyme activities found in the L58 callus (SOD: 0.42 ± 0.0398 nM (nanomole)/min/mg FW, POD: 4.1 ± 1.21) were compared with those found in the S18 callus (SOD: 0.24 ± 0.0471 nM/min/mg FW, POD: 1.2 ± 0.58) (Table 1). The enzymatic activities (SOD and POD) in the selected callus line L58 of *S. flavescens*, which showed the highest flavonoid metabolites content, were the highest in the eighth week.

### 2.7. Biomass Production and Growth Kinetics

**Figure 4:**
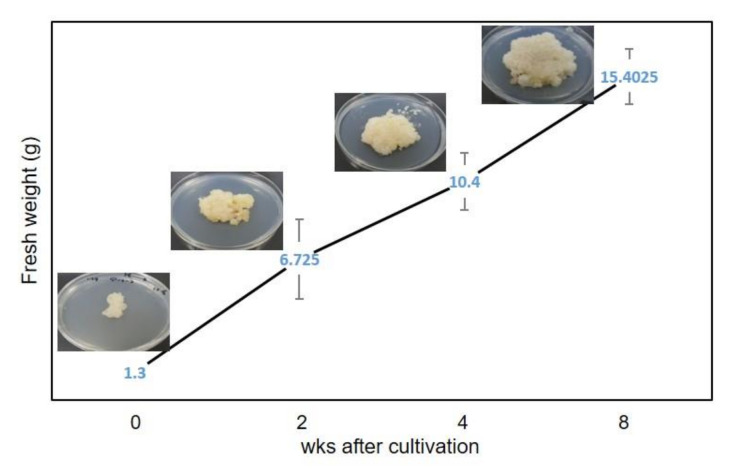
Fresh weight growth kinetics of *S. flavescens* callus (L58) cultures for 8 weeks. Values represent means ± standard error from triplicates.

The biomass accumulation of callus line L58, which contained the highest secondary metabolites, was increased by approximately 10 times when compared to the initial weight. Fresh weight of 1.3 g as starting material was inoculated on the medium, and fresh weight of 15 g was harvested after the eighth week of culture (Figure 4).

### 2.8. In Vitro Shoot Regeneration from the Callus

Induced calli from various treatments were continuously cultured to form shoots in 4-week subcultures. Interestingly, following 8 weeks of culture on media containing all combinations of TDZ and NAA, stem explants formed a compact, green, or yellow-green regenerative callus with buds and shoots. In contrast, leaf- or root-derived calli cultured on the same medium composition showed no shoot formation. There were 3–18 shoots formed from a single callus (Table 3). The shoots excised from the calli were rooted one month after transfer to the PGR-free MS medium. The rooting ratio of shoots was the highest in the 1.0 mg/L TDZ and 2.0 mg/L NAA (average = 75%, Table 3), and shoots were well grown with a thin cylindrical shape (2–6 cm in length and 1–2 mm in diameter) and were externally pale yellow.

## 3. Discussion

Currently, most plant resources for natural or Chinese medicine are taken from their natural habitats. This is not sustainable and negatively affects the natural environment over time. Furthermore, the “Nagoya Protocol” treaty regarding the conservation and sustainable use of biodiversity was signed in 2010 and was an important turning point for the indiscreet use of plant resources from around the world. Prerequisites for the commercialization of herbal medicinal products are mass production of the plant itself or the active ingredient. In the present study, we established optimal conditions for callus induction, isolation, and maintenance to accumulate high levels of the active ingredient, Maac, of *S. flavescens*. Callus formation of *S. flavescens* was influenced by the explant type, PGRs, and their interaction. The appearance of callus formation depended on the types and concentrations of the PGRs added, even for the same genus [30]. The in vitro production of secondary compounds in medicinal plants is possible due to variations in the culture conditions, including changes in the types and concentrations of PGRs [31,32]. Auxin and cytokinin are essential factors for the differentiation of the explant. The auxins initiate cell division and control the processes of growth and cell elongation because they induce the transcription of mRNA (microRNA) encoding proteins important for growth [33]. It is often reported that 2,4-D (2,4-dichlorophenoxyacetic acid) is the most suitable auxin for callus initiation and proliferation [34,35,36,37]. The cytokinins regulate many cellular processes, but the control of cell division is central to the growth and development of medicinal plants [33]. We found that the induction rate of calli, according to the cytokinin type, was the highest in the medium containing TDZ and was relatively low in that containing kinetin; while, for the auxins, the callus induction rate was highest in the medium containing picloram, followed by 2,4-D, and the lowest rate was in the medium containing NAA. Maximum callus induction in this study was observed in MS medium containing TDZ and picloram. Similar results were observed in the *Phlomis armeniaca* medicinal plant [38]. Our study demonstrated that the secondary metabolite Maac was present in significantly larger amounts in the undifferentiated cultured cells grown with 0.5 mg/L BA and 2.0 mg/L picloram when compared to the extracts of mother plant tissues.

The presence and accumulation of secondary metabolites is affected by the level of cellular differentiation and organization of the tissue [39]. Pasqua et al. [40] and Palacio et al. [31] suggested that the differentiation of specialized tissues is a prerequisite for the production of secondary metabolites. For example, the hypericin content in the callus was less than that found in in vitro-regenerated plantlets in comparative studies [40]. Cell cultures from *Digitalis lanata, Digitalis purpurea*, or *Azadirachta indica* did not induce secondary metabolites, such as cardenolide, digitoxin, or nimbin; however, after induction of organogenesis, these substances could be detected in differentiated tissue cultures [41,42,43]. In contrast, morphological differentiation is not a prerequisite, as the highest concentrations were detected in completely undifferentiated cells [44]. Karakas and Turker [38] demonstrated that the well-proliferated, green, and compact calli accumulated higher levels of caffeic acid and p-coumaric acid than the leaf tissue.

The number of subcultures (age of the callus) may also play an important role in the expression of secondary metabolites in vitro. The phenolic compounds and flavonoid contents in the superior cell line of *A. indica* have an inverse relationship with cell growth determined by callus weight [45], especially in media containing BA. The relationship between cell growth and the accumulation of secondary metabolites has been reported, although it is still not well understood [31,46]. Loredo-Carrillo et al. [46] suggested that a decrease in growth may be related to the use of energy from sucrose in the culture medium for the synthesis of secondary metabolites. In our study, calli with high weights had high accumulation of isoflavonoids. In addition, we were able to obtain cultures with high isoflavonoid levels even in the absence of light and without elicitation, while Loredo-Carrillo et al. [46] reported that the in vitro compounds were only produced in calli in the presence of light and polyethylene glycol.

When we aim fir mass production of bioactive secondary metabolites through callus culture, it may not be necessary to induce regeneration of in vitro shoots. Nevertheless, the development of in vitro propagation system is valuable because of the need to sustain natural resources due to global climate change. The genus *Sophora* shoot regeneration has been sparsely studied [47,48]. Zhao et al. [47] and Jana et al. [48] established a micro-propagation system *S. flavescens* and *Sophora tonkinensis*, respectively, using young stem node by optimizing PGRs’ combination conditions. Unlike our results, which showed high efficiency of shoot regeneration in a combination of 1.0 mg/L TDZ and 2.0 mg/L NAA, they reported that the best responses to shoot multiplication were observed in a combination of 8.88 μM 6-BA and 2.69 μM NAA for *S. flavescens* and 2.0 μM 2-isopentyladenine (2iP) for *S. tonkinensis*. In vitro shoot regeneration is highly influenced by the media formulations containing PGRs and other components. In addition, responses to them vary depending on the plant species, explant types, and culture environment. In this study, we developed optimal conditions for *S. flavescens’* shoot regeneration. We believe that it has provided basic results for future research to apply new biotechnologies on metabolic pathway engineering by revealing conditions that can induce plants from *S. flavescens’* callus, which is expected to contain bioactive compounds.

## 4. Materials and Methods

### 4.1. Study Materials

The seeds of *S. flavescens* were kindly provided by The Wild Plant Seed Bank of Korea. All macro and micronutrients for tissue culture media, sucrose, agar, and plant growth regulators (PGRs) were obtained from Duchefa (Haarlem, The Netherlands).

### 4.2. In Vitro Seed Germination

For germination, 52 seeds were sterilized with 10% (v (volume)/v) sodium hypochlorite solution for 15 min, placed on Murashige and Skoog (MS) [49] medium supplemented with 3% sucrose and solidified with 0.8% agar containing 2.0 mg/L gibberellin A_3_ (GA_3_), and incubated in a growth room at 25 ± 2 °C with a photoperiod of 16/8 h (light/dark). Germinated in vitro plants were propagated through subculture of one-month intervals and used in the experiments. All processes were carried out under sterile conditions using a laminar air-flow hood. In addition, the MS medium used was autoclaved at 121 °C for 15 min, and then the pH was adjusted to 5.8.

### 4.3. Callus Induction

Leaf, stem, and root explants of the in vitro-developed shoots were aseptically cut into 5 × 5 mm, 5~6 mm, and 7~8 mm pieces, respectively. They were then placed horizontally with their abaxial side down on MS medium supplemented with 0.0, 0.2, 1.0, and 2.0 mg/L of auxins (2,4-dichlorophenoxyacetic acid (2,4-D), picloram, and α-naphthalene acetic acid (NAA)) in combination with 0.0, 0.1, 0.5, and 1.0mg/L of cytokinins (kinetin, 6-benzyladenine (BA), and thidiazuron (TDZ)). A total of 100 combinations of media including 81 combinations consisting of three types of auxin and three types of cytokinins with three concentrations per each PGR, auxin-only, cytokinin-only, and without PGRs group were used. For each combination, 20 explants were cultured on each Petri dish (90 × 15 mm) with a replication of five Petri dishes. The percentage of callus induction was recorded after eight weeks of culture. Callus was subcultured every four weeks over a period of six months. The culture plates were maintained in the dark at 24 ± 1 °C.

For the statistical analysis, data were examined by analysis of variance (ANOVA) with a significance value of *p* < 0.05 using Microsoft Office Excel 2013 (Microsoft Corporation, Redmond, WA, USA), and Duncan’s multiple range test and Tukey’s honest significant difference test were used to compare the differences among the mean values.

### 4.4. Analysis of Flavonoid Metabolites by Ultra-High-Performance Liquid Chromatography (UHPLC)

Approximately 3 g each of selected calli, stem, young leaf, and root tissues from in vitro- or field-grown plants were lyophilized and pulverized to form a fine powder. Powder (1.25 mg/mL (w (weight)/v (volume)) from each sample was placed in flasks of methanol and extracted (repeated three times) for 20 min at room temperature in an ultrasonic extractor. The chromatographic separations of the samples were performed on an Acquity UHPLC system (Waters Co., Milford, MA, USA) with a BEH (Ethylene Bridged Hybrid) C18 column (100 mm, 2.1 mm, 1.7 μm, 130 Å, WT186002352) at 35 °C. The conditions were as follows: Solvent, acetonitrile; 0.4 mL/min flow rate; 5 μL injection; and 2540nm detection with a 4-nm bandwidth. Maac and other isoflavonoids (methoxy kurarinone (Kurari-OCH_3_), kushenol F (Kush F), kushenol E (Kush E), and kurarinone (Kurari)) were identified by comparing their ultraviolet spectra and the retention times of their peaks with those of authentic standards purchased from Sigma-Aldrich (St. Louis, MO, USA). Quantitative data were calculated on the basis of the peak area of each compound in the chromatograms at 254 nm. The contents were estimated as μg/g of dry weight of sample.

### 4.5. Total Phenolic Content

To determine the total phenolic content (TPC), Folin–Ciocalteu reagent was used according to the protocol of Slinkard and Singleton [50] with slight modifications. Briefly, the Folin–Ciocalteu reagent (90 μL) was added to each well containing 20 μL of the samples in 96-well plates. Then, sodium carbonate (90 μL) was added from the 6% stock solution to the wells, and plates were kept at room temperature for 90 min under normal room light. Gallic acid (1 mg/mL) and methanol (20 μL) were used as positive and negative controls, respectively. The absorbance was measured at 725 nm by using a UV-VIS spectrophotometer (UV-160A, Shimadzu Ltd, Tokyo, Japan). The calibration curve (0–50 μg/mL, R^2^ = 0.968) was plotted by using gallic acid as the standard, and the TPC was expressed as gallic acid equivalents (GAE)/g of dry weight. TPC was measured by using the following formula and estimated in mg GAE/L:

Total phenolic production mg/L = Dry weight (g/L) × Total phenolic content (mg/g)(1)

### 4.6. Total Flavonoid Content

The total flavonoid content (TFC) concentration was quantified using the colorimetric assay method from [51] with slight modification. Briefly, 50 μL of crude extract (1 mg/mL ethanol) was dissolved in methanol to a total volume of 1 mL. The solution was mixed with 4 mL of distilled water and then we added 0.3 mL of 5% NaNO_2_ solution. Then, 0.3 mL of 10% AlCl_3_ solution was added after 5 min of incubation, and the mixture was allowed to stand for 6 min at room temperature. Then, 2 mL of 1 mol/L NaOH solution was added, and the final volume of the mixture was brought to 10 mL with double-distilled water. The mixture was allowed to stand for 15 min, and absorbance was measured at 510 nm. The TFC was calculated from a calibration curve (0–40 μg/mL, R^2^ = 0.998), which was plotted by using quercetin as the standard. The TFC was expressed as mg quercetin equivalent (QE)/g of dry weight. Total flavonoid production (TFP) was calculated by using the following formula and expressed in mg QE/L:

TFP mg/L = Dry weight (g/L) × TFC (mg/g)(2)

### 4.7. DPPH Radical Scavenging Activity Determination

The antioxidant activity of *S. flavescens* callus extracts was assessed on the basis of the free radical scavenging effect of 2,2-diphenyl-1-picrylhydrazyl (DPPH) according to the method described by Tiwari et al. [52] with slight modification. Ten grams (fresh weight (FW)) from selected calli showing high maackiain content was harvested and completely dried in a dry oven for 48 h at 50 °C. A mixture of dry sample and methanol (1:10 (w/v)) was incubated at 50 °C overnight in a water bath. A 100 μmol/L solution of DPPH was added to the methanol extracts in varying concentrations (1.0, 2.0, 3.0, 4.0, and 5.0 (mg/mL)). The solutions were kept in the dark for 20 min to complete the reaction, and then the absorbance was measured at 517 nm. Ascorbic acid, in a series of concentrations, was used as the reference material. All tests were performed in triplicate. The percentages of DPPH radical scavenging activities of *S. flavescens* callus extracts were calculated using the following formula [53]:

DPPH (%) activity = [(A0 − A1) / A0] × 100(3)

where A0 is the absorbance of the blank and A1 is the absorbance of the sample.

### 4.8. Peroxidase Activity

Samples were extracted to determine peroxidase (POD) activity using the protocol of Nayyar and Gupta [54] with slight modification. Briefly, 100 mg of the fresh sample was homogenized with 1 mL potassium phosphate buffer (50 mM, pH 7) containing 1% PVP (polyvinylpolypyrrolidone) and centrifuged at 15,000 rpm for 30 min. The supernatant was retained for further analysis. The reaction mixture for POD activity was prepared by combining 40 μL potassium phosphate buffer (50 mM; pH 7), 20 μL guaiacol (100 mM; 10×), 20 μL fresh sample extract, 100 μL dH_2_O, and 20 μL H_2_O_2_ ((27.5 mM; 10×) [55]). The control was carried out by applying equal amounts of all the reagents except the sample extract. After the 20-s incubation period, the absorbance was recorded at 470 nm using a Thermo Scientific Multiskan GO, and the enzymatic activity was measured using the following formula:

Absorbance = E (Extinction coefficient, 6.39/mM/cm) × L (Length of wall, 0.25 cm) × C (enzyme concentration, value calculated in nM/min/mg FW)(4)

### 4.9. Superoxide Dismutase Activity

Superoxide dismutase (SOD) activity was determined using an assay kit (Cell Biolabs. Inc, San Diego, CA, USA) according to the protocol provided by the manufacturer. Briefly, the activity was obtained by mixing 20 μL of the reaction mixture with 60 μL of fresh sample extract. The same mixture excluding extract samples was run as a control. After a 2-h incubation period under fluorescent light, the absorbance was measured at 490 nm with a microplate reader (Thermo Scientific Multiskan GO). In order to measure the enzymatic activity, the same formula was used as for POD.

### 4.10. In Vitro Shoot Regeneration

For shoot regeneration, induced calli were transferred to MS medium containing different concentrations and combinations of both TDZ (0.1, 0.5, and 1.0 mg/L) and NAA (0.2, 1.0, and 2.0 mg/L). Cultures were maintained in a growth chamber at 24 ± 1 °C with a 16-h photoperiod (light intensity of 40 μmol/m^2^/s^1^ provided by fluorescent tubes). The best combination with the highest shoot regeneration frequency was chosen, and regenerated shoots were grown on PGR-free media.

## 5. Conclusions

We identified that leaf-derived calli grown on MS medium containing 2.0 mg/L picloram and 0.5 mg/L BA produced approximately 10-fold higher Maac than wild-type plants. Findings of our study will be helpful for increasing the mass production of Maac in vitro for commercialization.

## Figures and Tables

**Table 1:** Quantitative analysis of TPC, TFC, DPPH, SOD, and POD in *Sophora flavescens* callus and explant (n = 3 biological replicates).

	Callus Type	TPC (mg GAE/g FW)	TFC (mg QE/g FW)	DPPH (%)	SOD (nM/min/mgFW)	POD (nM/min/mgFW)
Root *	-	7.82 ± 0.52a	9.75 ± 0.18a	84.00 ± 6.56a	0.71 ± 0.04a	5.60 ± 0.43a
Root (in vitro) **	-	5.09 ± 0.28bc	5.80 ± 0.39b	70.67 ± 8.02ab	0.68 ± 0.04a	5.07 ± 0.29a
L58 (0.5 mg/L BA + 2.0 mg/L picloram)	LY/JS	6.00 ± 0.30b	5.40 ± 0.56b	62.67 ± 7.09b	0.42 ± 0.04b	4.10 ± 1.31ab
R76 (0.5 mg/L TDZ + 0.2 mg/L 2,4-D)	GY/F	4.93 ± 0.59bc	3.22 ± 0.67c	57.00 ± 7.00b	0.38 ± 0.06bc	2.37 ± 0.23bc
S18 (1.0 mg/L Picloram)	Y/F	3.99 ± 0.28c	4.96 ± 0.33b	32.00 ± 5.29c	0.24 ± 0.04c	1.22 ± 0.59c
S55 (0.1 mg/L BA + 2.0 mg/L picloram)	Y/JS	5.93 ± 0.44b	3.61 ± 0.17c	55.00 ± 4.58b	0.25 ± 0.07c	3.13 ± 0.47b
S88 (0.5 mg/L TDZ + 2.0 mg/L picloram)	Y/JS	5.31 ± 1.28bc	3.91 ± 0.19c	53.00 ± 8.54b	0.33 ± 0.04bc	2.67 ± 0.55bc

Different letters indicate significant difference at the 5% level by Tukey’s honest significant difference test. * Indicates that the materials come from field-grown S. flavescens root; ** indicates that the materials come from in vitro S. flavescens root (μg/g dry weight). Color: Yellowish (Y), light yellow (LY), green yellow (GY), white (W). Texture: Friable (F), juicy and sticky (JS). TPC: Total phenolic content, expressed in gallic acid equivalent (mg GAE/g). TFC: Total flavonoid content, expressed in quercetin equivalent (mg QE/g). DPPH: 2,2-diphenyl-1-picrylhydrazyl. SOD: Superoxide dismutase. POD: Peroxidase.

**Table 2:** Quantitative analysis of maackiain and other isoflavonoids from UHPLC in *S. flavescens* callus and explant (n = 3 biological replicates).

	Maackiain	Kurari-Methoxy	Kush F	Kurari	Kush E
Root *	145.9a	468.6b	1621.4d	299.1a	0
Root (in vitro) **	123.3a	69.2a	933.1c	781.3b	0
L58	1580.6d	0	229.1b	0	0
R76	653.7c	0	0	0	0
S18	428.3b	0	0	0	0
S55	592.4c	0	182.6b	0	0
S88	395.9b	0	14.8a	0	0

* Indicates that the materials came from field-grown S. flavescens root; ** indicates that the materials came from in vitro S. flavescens root (μg/g dry weight). Different letters indicate significant difference at the 5% level by Duncan’s multiple range test. UHPLC: Ultra-High Performance Liquid Chromatography. Kush F: Kushenol F. Kurari: Kurarinone. Kush E: Kushenol E.

**Table 3:** Shoot regeneration through organogenesis in *Sophora flavescens* callus induced from stem on MS medium treated with the TDZ (thidiazuron) and NAA (α-Naphthalene acetic acid) combinations shown in Table 1. They were incubated in a growth room at 24 ± 1 °C, 16/8 h (light/dark).

Plant Growth Regulators (mg/L)		Shoot Numbers Per Callus	Rooting Rates (%)
TDZ	NAA		
0.1	0.2	4.0 ± 0.68a	25.0 ± 4.52b
	1.0	3.1 ± 0.90a	66.0 ± 14.6d
	2.0	9.0 ± 1.08c	11.0 ± 1.37a
0.5	0.2	6.0 ± 0.97b	66.0 ± 9.88d
	1.0	4.1 ± 0.58a	25.0 ± 7.72b
	2.0	18.0 ± 1.53d	16.0 ± 8.10a
1.0	0.2	6.0 ± 1.18b	66.7 ± 5.06d
	1.0	3.0 ± 0.97a	33.3 ± 12.48c
	2.0	4.2 ± 0.64a	75.0 ± 6.27e

Values represent the mean ± standard deviation. Values within the same column followed by the same letter are not significantly different according to the least significance at *p* < 0.05 (Duncan 1955). Each treatment consisted of three replications of 100 explants each. All data were collected 8 weeks after being inoculated in the medium supplemented with PGRs (plant growth regulators).

## Abbreviations

GA_3_ (Gibberellin A_3_); 2,4-D (2,4-dichlorophenoxyacetic acid); NAA (α-Naphthalene acetic acid); BA (6-benzyladenine); TDZ (Thidiazuron); PGRs (Plant growth regulators); Maac (Maackiain); Kurari-OCH_3_ (Methoxy kurarinone); Kush F (Kushenol F); Kush E (Kushenol E); Kurari (Kurarinone); UHPLC (Ultra-High Performance Liquid Chromatography).
